# Using electrocardiogram electrodes to monitor skin impedance spectroscopic response when skin is subjected to sustained static pressure

**DOI:** 10.1002/ski2.225

**Published:** 2023-03-06

**Authors:** Emily J. Owen, Hollie Hathaway, Bronwen Lafferty, A. Toby A. Jenkins

**Affiliations:** ^1^ Department of Chemistry University of Bath Bath UK; ^2^ Advanced Wound Care Convatec Ltd. Deeside UK

## Abstract

**Background:**

Impedance spectroscopy is a non‐invasive technique which can be used to monitor skin barrier function, with potential applications in early‐stage pressure ulcer detection. This paper describes how changes in skin impedance, due to mechanical damage of the stratum corneum by tape stripping or applied pressure, can be straightforwardly measured using commercial electrocardiogram electrodes and a relatively low‐cost impedance analyser. Two models of pressure injury were studied, an ex vivo porcine and in vivo human skin model.

**Objectives:**

Determine whether impedance spectroscopy may have potential utility in measuring the effect on skin of applied pressure on early‐stage pressure injury.

**Methods:**

Two models were utilized to measure the effect of pressure. *Porcine model*: 0, 7.5, 15 or 22.5 mmHg of pressure was applied for up to 24 h (*N* = 4) and monitored at various time intervals. *Human Model:* 88 mmHg of pressure was applied for four sets of three‐minute intervals (*N* = 13) and post‐pressure recovery was monitored for 4 h. For each model, skin impedance was monitored at 0.1 Hz–50 kHz using disposable Ag/AgCl electrodes. The data was analysed using Ordinary One‐Way Analysis of Variance.

**Results:**

*Porcine model*: after 24 h, the impedance of pressure‐loaded skin was significantly reduced compared to the non‐loaded control group (*p* ≤ 0.0001); this reduction in impedance was proportional to the degree of mechanical loading. Histology images of skin cross‐sections provided qualitative evidence that the epidermis was structurally compromised by pressure. *Human Model:* the response of healthy skin to applied pressure displayed inter‐variation. Participants with a significant change in skin impedance (*p* ≤ 0.01) also demonstrated signs of erythema.

**Conclusions:**

This study suggests that using impedance spectroscopy to measure skin (stratum corneum) resistance may have utility in giving early warning of skin pressure injury prior to clinical symptoms, with a good correlation between observed erythema and reduction in skin resistance. Further work should be initiated on patients at risk of pressure injury to improve intervention strategies, including in darker skin tones where early‐stage pressure injuries may not be visually distinct.

## Graphical abstract

The utility of impedance spectroscopy in monitoring pressure injuries was assessed, using an ex vivo porcine model and in vivo human model. This technique could support visual assessment of pressure ulcers and could have particular utility in assessing patients with darker skin tones, where early‐stage symptoms are not always distinct.

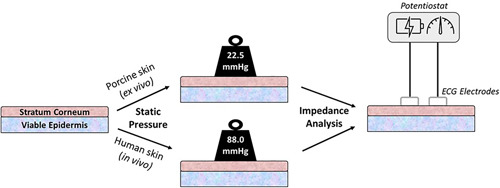

1

**What's already known about this topic?**

- Non‐blanchable erythema is a key indicator of early‐stage pressure injury. However, there is low inter‐observer reliability associated with the European Pressure Ulcer Advisory Panel (EPUAP) classification system due to its reliance on visual assessment; diagnosis is particularly challenging on darker skin tones. Impedance spectroscopy is becoming an increasingly accepted method of monitoring skin barrier function. There is evidence to suggest the utility of impedance spectroscopy to monitor pressure ulcer formation.

**What does this study add?**

- Applying static pressure to ex vivo porcine skin and in vivo human skin results in a significant drop in impedance; this may be used to give an early warning of pressure injury prior to ulceration. In the in vivo study, it was found that visible erythema was consistently accompanied by a drop in skin impedance. This could improve assessment of early‐stage pressure injury in darker skin tones.

**What is the translational message?**

- Impedance spectroscopy is a useful tool which can be translated into clinical use; it could be used routinely as part of pressure ulcer assessment to support clinical observations. Whilst some patients require resource‐intensive intervention strategies to prevent pressure injury, others may have a higher tolerance. Impedance spectroscopy could therefore allow resources to be focussed where necessary rather than implementing a generic management plan for all patients.

## 2 INTRODUCTION

### 2.1 Correlating skin impedance to skin barrier function

The skin provides a protective barrier from external insults, composed of three principal layers: epidermis, dermis and subcutaneous tissue.^1^ The outermost layer of the epidermis, known as the stratum corneum, consists of 15‐20 layers of dead, enucleated corneocytes.^2^ The stratum corneum is responsible for providing a barrier in transdermal transport.^3^ Stratum corneum properties, including thickness and water content, provide information on skin barrier function. Currently, the most conventional measure of skin barrier integrity is transepidermal water loss (TEWL) but differing external environments can cause significant variability in measurements.^4^, ^5^

Electrical Impedance Spectroscopy (EIS) is an efficient technique used to non‐invasively characterize tissue; pathophysiological alterations to tissue cause changes in the flow of electrical current through the tissue under question.^6^, ^7^ EIS has advantages over other tissue characterizing techniques; Bioelectrical Impedance Analysis typically uses a single frequency of 50 kHz to estimate body composition whilst EIS involves multifrequency impedance analysis to provide more information on tissue properties.^7^ Skin impedance is predominately measured using low‐frequency EIS, frequencies of 1 kHz and below are dominated by the stratum corneum impedance and can therefore be used to monitor skin barrier function.^3^ White *et al.* reported changes in skin impedance caused by irreversible mechanical damage of ex vivo human skin via needle puncture.^8^ Furthermore, Rinaldi *et al.* found that the impedance of murine skin is altered when exposed to proteolytic enzymes.^5^ EIS has demonstrated complementarity with TEWL when monitoring stratum corneum integrity.^9^

In the present investigation, the serial tape stripping technique was used to experimentally induce impaired skin barrier function; the mechanical stress debrides the stratum corneum.^10^, ^11^, ^12^ The gradual removal of the highly resistive cells was monitored using skin impedance. Electrocardiogram (ECG) electrodes (pre‐gelled Ag/AgCl electrodes) were employed because they have better skin contact than standard dry electrodes and are not associated with resource‐intensive fabrication.^3^, ^13^

### 2.2 Monitoring pressure injury with skin impedance

According to the National Pressure Ulcer Advisory Panel and EPUAP, pressure ulcers are “localized injury to the skin and/or underlying tissue usually over a bony prominence, as a result of pressure, or pressure in combination with shear”.^14^ Pressure ulcer treatment places significant financial strain on the health and social care system in the UK.^15^ It has been estimated that the current daily cost to the National Health Service associated with pressure ulcer care is greater than £3.8 million.^16^ Sustained pressure causes tissue deformation via two main pathways, each contributing to the risk of pressure ulceration. Firstly, the pressure site has a reduction in perfusion which can lead to localized ischaemia and, in many cases, reperfusion injury (once the pressure is removed). Secondly, the static pressure itself is believed to cause direct cellular deformation by compromising the structural integrity of the cytoskeleton and plasma membrane.^17^, ^18^ Whilst absolute measures have not been established, the literature suggests that there is an inverse relationship between the duration and magnitude of pressure applied to tissue which will ultimately lead to skin degradation.^19^

There is currently no tool which can be used to identify individuals displaying early‐stage pressure injuries. An individual's internal response to a specified magnitude/duration of loading is inter‐variable.^20^ Furthermore, there is low inter‐observer reliability associated with the EPUAP classification system.^21^ For example, non‐blanchable erythema is one of the key indicators of early‐stage pressure ulcers but darker skin tones may only present with dicolouration.^22^ It is crucial to develop tools which can assess the extent of pressure injury without reliance on visible inspection. Swisher *et al.* used an in vivo murine model to detect pressure‐induced damage via impedance spectroscopy in cases which were not apparent by visual inspection. This was carried out using resource‐intensive dry electrodes and the impedance data was only shown at select frequency intervals.^23^ However, in this present paper a simplified model using cost‐effective ECG electrodes was employed and the multi‐frequency impedance data was fitted to an equivalent circuit model. The ex vivo porcine model detailed in this work aims to explore the effect of cellular deformation alone caused by pressure. Next, an in vivo human model was used to monitor the response of healthy intact skin to pressure.

## 3 MATERIALS AND METHODS

### 3.1 General materials

The PalmSens4 potentiostat was from PalmSens, The Netherlands. The MoistureMeterSC (stratum corneum moisture measurements) was sourced from Delfin Technologies, Finland. Red Dot 3M Electrodes and the A&D Medical UM‐102B Sphygmomanometer were sourced from Medisave, UK. Microtome blades, frosted microscope slides and 4% Paraformaldehyde (PFA) solution in Phosphate Buffered Saline (PBS) were purchased from Fisher Scientific, UK. Phosphate Buffered Saline tablets (pH 7.4), HistoChoice, concentrated HCl and DPX mounting medium were obtained from Sigma‐Aldrich, UK. The Remington G3 Graphite Razor was from Boots, UK. Haematoxylin (modified Mayer's solution), Bluing reagent and Eosin Y solution were from Abcam, UK. The porcine skin was sourced from a local abattoir, UK.

### 3.2 Methodology

#### 3.2.1 Porcine skin preparation

Dorsal ex vivo porcine skin (Oxford Sandy and Black) was stored at −20 °C. Skin was thawed for 60 min in a humidity cabinet (Memmert HCP50) at 32 °C and 50% relative humidity. The porcine hair was trimmed to 1.5 mm using a Remington G3 Graphite razor, washed in PBS and air dried for 15 min. The full thickness skin samples, still attached to the subcutaneous fat, were approximately 1 cm thick.

#### 3.2.2 Electrode set‐up

Skin impedance was measured using a two‐electrode method; one ECG electrode connected to the working electrode and the other ECG electrode connected to the counter electrode, combined with the reference electrode. The chosen electrodes were Red Dot 3M (Figure S1, ESI). For porcine skin, measurements took place in a plastic petri‐dish.

#### 3.2.3 Impedance measurement parameters and data fitting

Skin impedance was measured using a PalmSens4 handheld potentiostat (PalmSens, The Netherlands). A 10 mV amplitude sinusoidal potential was applied, with frequency swept from 50 kHz to 1 Hz with 10 points being measured per decade. Data was fitted to the relevant equivalent circuits using the PSTrace 5.8 software.

#### 3.2.4 Histology

Samples were fixed in 4% PFA at 4 °C for 24 h and stored in 70% ethanol. Samples were paraffin‐embedded using an automatic tissue processor (Leica, UK). Tissues were sectioned at 5 μm and stained using Haematoxylin and Eosin.

#### 3.2.5 Tape stripping of ex vivo porcine skin

A strip of Sellotape was applied evenly to the desired skin region, using mild pressure. The strip was ripped off slowly, alternating the direction by 180° each time. Each skin site was tape stripped 110 times.

#### 3.2.6 Static applied pressure on ex vivo porcine skin

Static pressure of 7.5, 15.0 or 22.5 mmHg was applied to ex vivo porcine skin for up to 24 h, incubated at 32 °C and 50% relative humidity. The mechanical loads were only removed during the measurement period, involving the use of new ECG electrodes each time.

#### 3.2.7 Static applied pressure on in vivo human skin

The study, involving thirteen mixed‐sex healthy volunteers, was approved by the Bath Research Ethics Approval Committee (EP 22 001). Inclusion criteria: aged 16 or over and able to give informed consent. Exclusion criteria: irritated/abrased skin and dermatological conditions at the measurement sites. The ventral forearm test sites were wiped with water, gently dried with paper towels and left for 10 min. Baseline stratum corneum moisture, TEWL and skin impedance of the ventral forearm sites was measured. The pressure cuff was placed over the ECG electrodes. For the left arm, 88 mmHg of pressure was applied for 3 min, followed by rest for 3 min. This was repeated three times. The right arm served as a control – electrodes were applied but there was no mechanical loading. After pressure cuff removal, the skin was wiped with water and dried again. Skin impedance, stratum corneum moisture and TEWL were re‐measured at 10, 60, 120, 180 and 240 min after pressure cuff removal (using new ECG electrodes each time).

## 4 RESULTS AND DISCUSSION

### 4.1 Change in skin impedance with partial stratum corneum debridement

An ex vivo porcine skin model was regarded as a close substitute for human skin in terms of structure, thickness, pigmentation, lipid composition, collagen and hair follicle content.^24^ The changes in skin impedance were measured prior to and throughout serial tape stripping.

#### 4.1.1 Impedance measurement fit and frequency range

**FIGURE 1:**
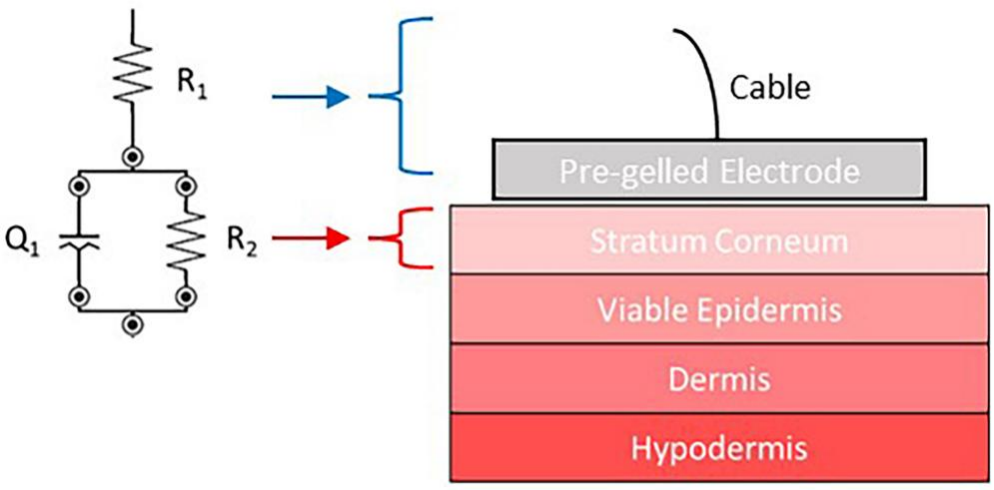
Skin model and electrical circuit model of skin R_1_(R_2_Q). R_1_ relates to the cable and pre‐gelled electrode resistance whilst (R_2_Q) relates to the stratum corneum.

Two main types of equivalent circuit models of skin are proposed in the literature; Resistor‐Capacitor (RC) layered models and constant phase element (CPE) models.^3^, ^25^ In RC layered models each parallel resistor and capacitor correspond to a distinct layer of skin but do not account for the heterogenicity of skin. To address this issue, the capacitor can be replaced with a CPE, as first reported by Cole in 1940.^3^, ^13^, ^25^, ^26^ CPEs model a distribution of capacitive elements, Z_Q_ = (ωC)^−n^, where *n* = 1 for a simple capacitance and *n* = 0.8 for human skin.^3^ In this work, it is proposed that the impedance of intact skin is dominated by the stratum corneum at frequencies between 50 kHz and 1 Hz. The data was fitted to an R_1_(R_2_Q) circuit model (Figure 1); the first series resistance (R_1_) models cable resistance and the parallel resistor and CPE (R_2_Q_1_) model the stratum corneum properties. With tape stripping, and subsequent damage to the stratum corneum, changes in skin barrier function can be monitored based on the circuit elements.

**FIGURE 2:**
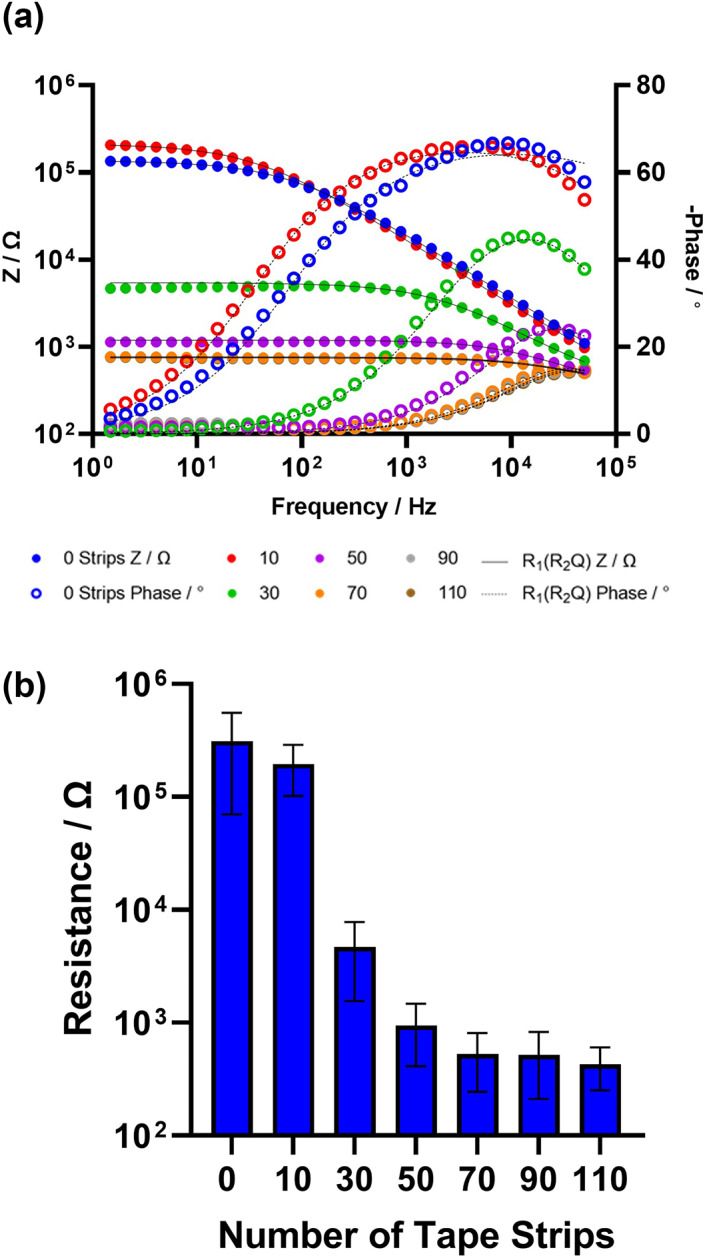
Skin impedance as a function of the number of tape strip events (a); left axis = impedance magnitude ‘Z’ and right axis = ‐phase, versus applied frequency of applied voltage. Data fit, R_1_(R_2_Q), is shown as the continuous line (Z) and dashed line (phase). Change in fitted resistance, R_2_, of an R_1_(R_2_Q) circuit is shown as a function of the number of tape strip events (b).

The change in total skin impedance and phase is shown in a Bode plot, as a function of the number of individual tape strip events (Figure 2a). The multi‐frequency impedance spectra of 0–110 strips were fitted to the proposed circuit model R_1_(R_2_Q) between 50 kHz and 1.5 Hz. There was little change in R_1_ as a function of tape stripping and the physical meaning of constant phase elements is unclear (Table S1, ESI).^27^ Therefore, the stratum corneum resistance (R_2_) was found to be an appropriate component to quantify the changes in impedance (Figure 2b). R_2_ decreased by three orders of magnitude between 0 and 70 tape strips. No additional decrease in resistance was found between 70 and 110 tape strips; deeper stratum corneum layers have stronger cohesive forces compared to the uppermost layers which are approaching desquamation and more readily detached.^28^, ^29^ Progressive removal of the stratum corneum and consequent reduction in skin barrier function correlated with a reduction in impedance.^5^, ^9^ The skin impedance protocol employed here, using ECG electrodes, can monitor stratum corneum integrity and therefore skin barrier function.

#### 4.1.2 Histology

**FIGURE 3:**
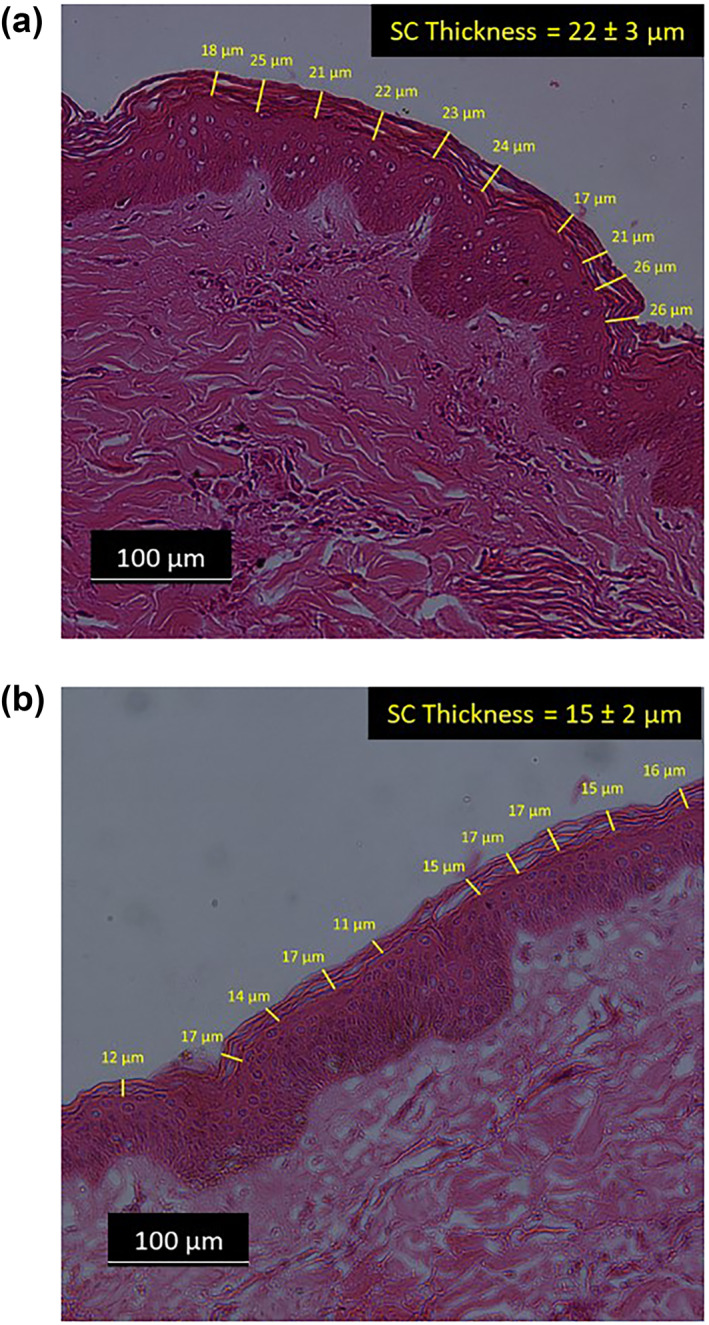
Histology images of ex vivo porcine skin cross‐section prior to tape stripping (a) and after 110 tape strips (b). The tissue samples were stained using Haematoxylin and Eosin staining.

To confirm that the stratum corneum was thinned by tape stripping, histology of the skin was carried out prior to stripping and after 110 tape strips (Figure 3). After stripping, the layers of cells in the stratum corneum were sparse and thin (Figure 3b: 15 μm) compared to the baseline (Figure 3a: 22 μm). It is therefore reasonable to assume that any changes in impedance are predominately due to superficial changes to stratum corneum integrity rather than deeper in the epidermis/dermis.

### 4.2 The effect of static pressure on ex vivo porcine skin

In 3.1, it was shown that changes in skin barrier function can be measured using impedance spectroscopy via ECG electrodes. Next, static pressure (0–22.5 mmHg) was applied to ex vivo porcine skin for up to 24 h to demonstrate the utility of this simple technique to detect early‐stage pressure injury. Whilst the ex vivo pressure model does not portray the damage cause by ischaemia‐reperfusion injury, it is useful in reflecting the cellular deformation caused by mechanical stress.

#### 4.2.1 Impedance measurement

**FIGURE 4:**
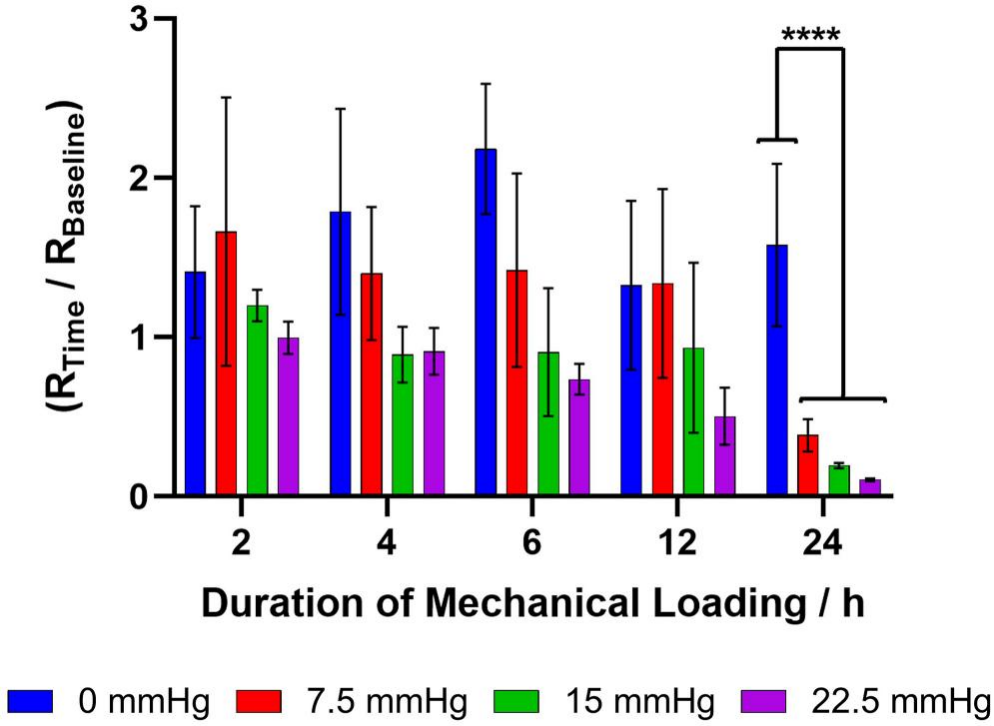
Impedance of ex vivo porcine skin, subjected to 0, 7.5, 15 or 22.5 mmHg of pressure, for 24 h. Fitted resistance ‘R_2_’, from an R_1_(R_2_Q) circuit model, was normalized with respect to the baseline (R_Time_/R_Baseline_). Error bars represent the standard deviation of four independent replicates, analysed using an Ordinary One‐Way Analysis of Variance: *p* ≤ 0.05 (*) and *p* ≤ 0.0001 (****).

The impedance data was fitted to an R_1_(R_2_Q) equivalent circuit model, between 50 kHz and 1 Hz. To account for natural biological variation between skin sites the resistance was normalized; the R_2_ resistance at each time interval is shown as a fraction of its original baseline resistance (Figure 4) (Table S2, ESI provides the raw data).

The pressure (0–22.5 mmHg) applied to the skin did not significantly impact R_2_ between 2 and 12 h after introducing the mechanical load (Figure 4). After 24 h, the R_2_ of mechanically loaded skin had a significant ten‐fold decrease (*p* ≤ 0.0001), whilst the non‐loaded control was unchanged. The reduction in R_2_ appears to be roughly proportional to the magnitude of pressure placed on the skin, although the difference is not statistically significant. Over time, the sustained static pressure structurally compromises the stratum corneum which ceases to provide an effective barrier to external insults. The established skin impedance protocol can monitor the decline in skin barrier function, with response to pressure, using readily available ECG electrodes.

#### 4.2.2 Histology

**FIGURE 5:**
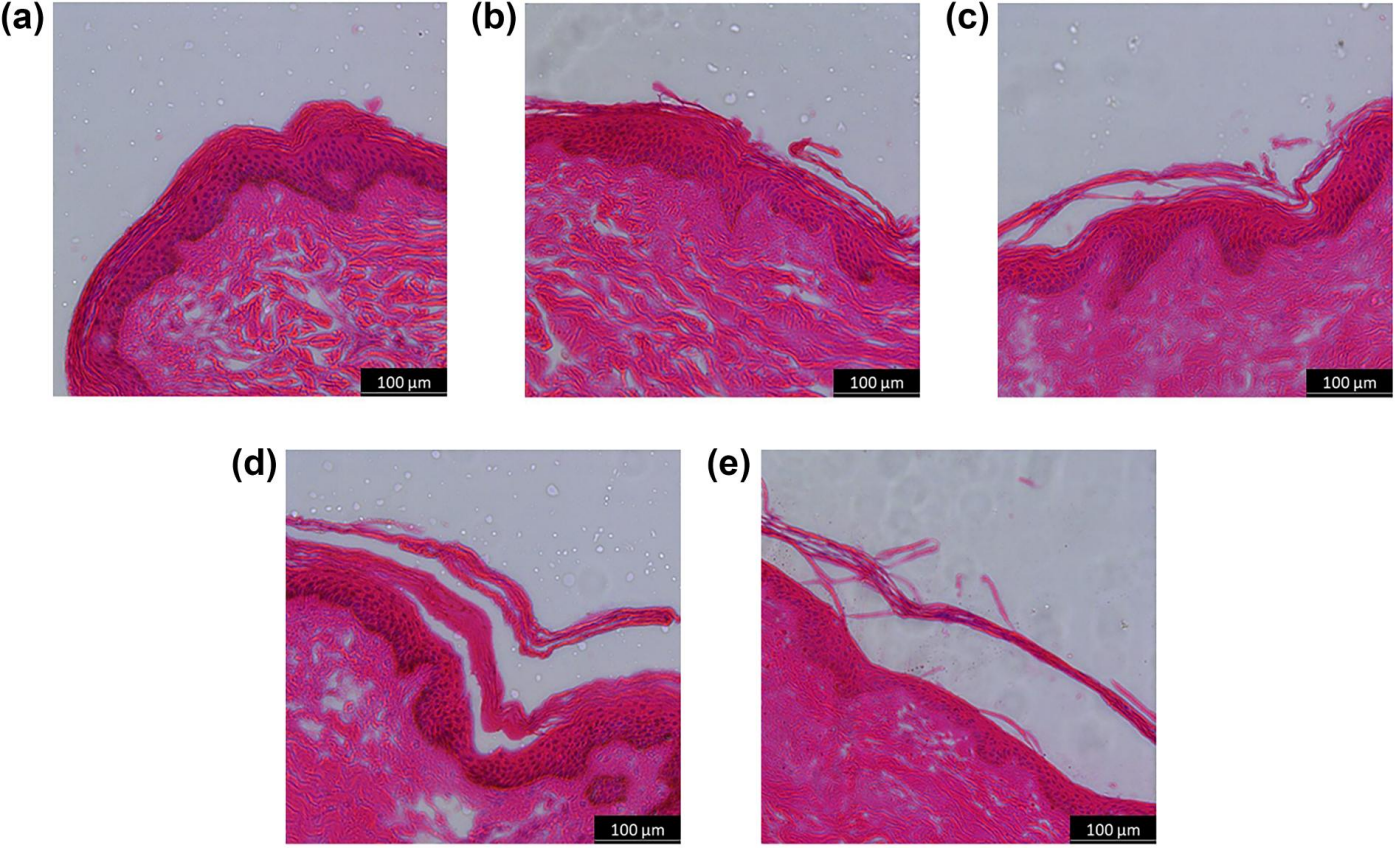
Histology images of ex vivo porcine skin cross‐section prior to applying static pressure (a) and after 24 h of sustained pressure: 0 mmHg (b), 7.5 mmHg (c), 15 mmHg (d) or 22.5 mmHg (e). The tissue samples were stained using haematoxylin and eosin staining.

Histological analysis of the pressure‐loaded skin confirms that the mechanical loading conditions caused cellular damage (Figure 5). Prior to mechanical loading, the porcine skin cross‐section was intact (Figure 5a). After 24 h, the control was unchanged (Figure 5b) whilst there is clear maceration of the skin and detachment of the stratum corneum from the rest of the epidermis when static pressure is applied (Figure 5c–e). This is in line with clinical presentations of superficial pressure injury, which can eventually progress to deep tissue ulceration.^19^

#### 4.2.3 Stratum corneum moisture measurement

**FIGURE 6:**
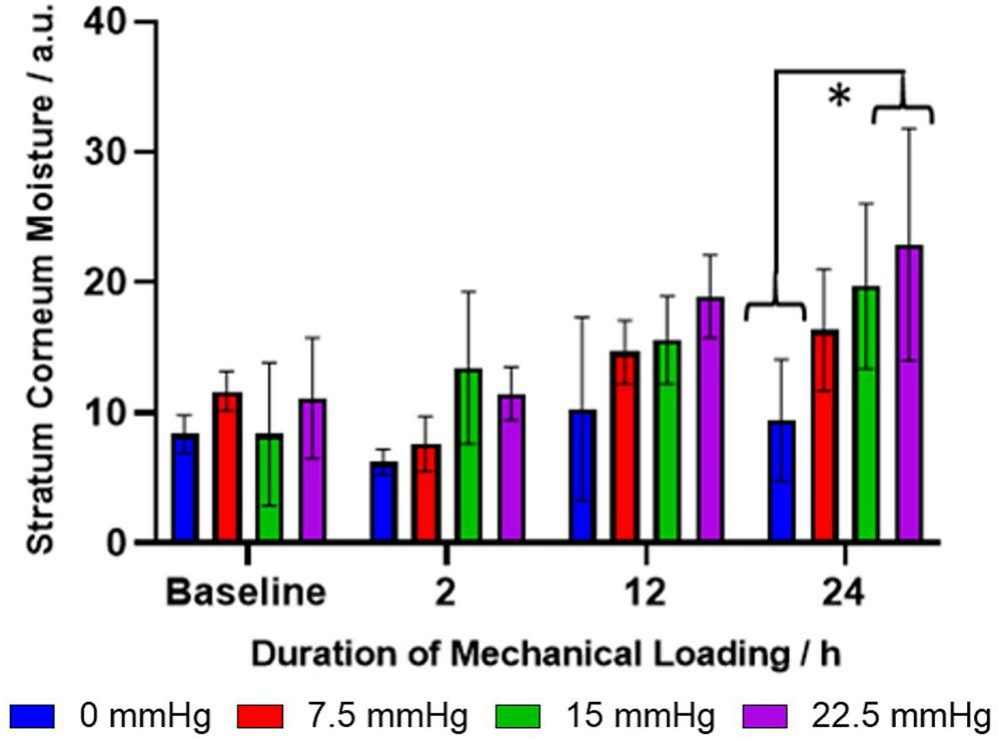
Effect of applied pressure and duration of applied pressure on stratum corneum moisture content of ex vivo porcine skin. Error bars represent the standard deviation of four independent replicates, analysed using an Ordinary One‐Way Analysis of Variance: *p* ≤ 0.05 (*).

Stratum corneum moisture, measured using a single frequency (1.25 MHz) electrode, is commonly used to assess skin barrier function.^30^, ^31^ Prior to the application of static pressure, the stratum corneum moisture of each skin group ranged between 8–12 a.u (Figure 6). After 24 h of mechanical loading, the non‐loaded skin remained within the initial baseline range. However, where skin was subjected to 22.5 mmHg of static pressure the moisture increased significantly to 23 a.u. (*p* ≤ 0.05). The increase in stratum corneum moisture resulting from sustained static pressure is indicative of epidermal maceration and superficial skin damage, supporting the impedance data (Figure 4).

### 4.3 The effect of static pressure on in vivo human skin

The *ex‐vivo* study of applied pressure on porcine skin (3.2) suggested that impedance spectroscopy is sensitive to damage caused by such application. Impedance spectroscopy might have a potential clinical utility in identifying patients who are suffering from early‐stage pressure injury before skin damage is observed. To test this hypothesis, the ventral forearms of 13 healthy volunteers were subjected to four sets of three‐minutes of static pressure (88 mHg) and three‐minutes of rest, via a blood pressure measurement cuff. The applied pressure of 88 mmHg was chosen because it did not cause excessive discomfort to the participants. Skin recovery was monitored using impedance spectroscopy, up to 240 min post‐pressure, and evidence of skin erythema was recorded (Figure S2, ESI).

#### 4.3.1 Impedance measurement

Results from the study were broadly grouped into three categories (Table 1).

**TABLE 1:** Change in skin impedance and evidence of erythema of 13 human participants after applying static pressure to the ventral forearms.

Group	Impedance change	Erythema?	Count
1	Skin resistance decreased irrespective of magnitude of applied pressure including on zero pressure controls.	Yes.	2
2	Skin resistance significant decrease on pressure arm (*p* ≤ 0.01); no change on zero pressure controls.	Yes ‐ pressure site only.	3
3	No skin resistance change.	No.	8

**FIGURE 7:**
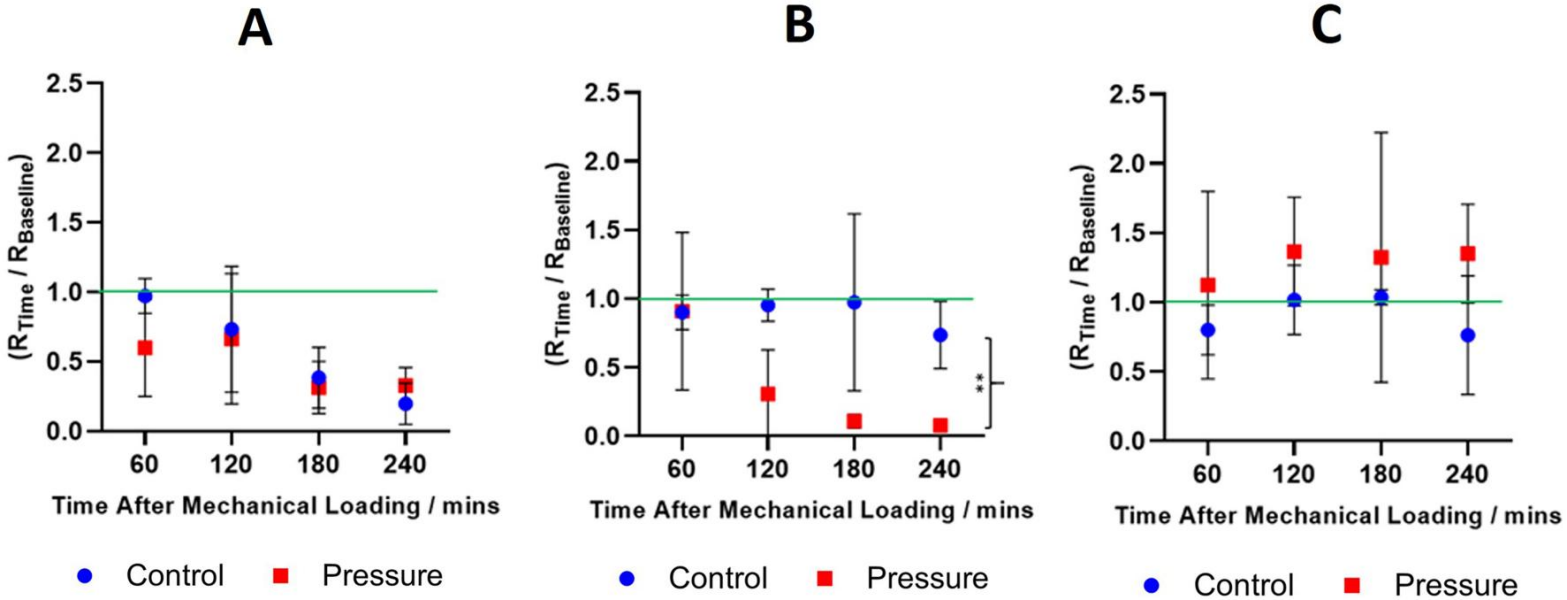
Impedance data for in vivo human ventral forearm skin of three participants representative of the three response groups (Group 1 (a), Group 2 (b), Group 3 (c), up to 240 min after applying 88 mmHg of static pressure. Fitted resistance ‘R_2_’, from an R_1_(R_2_Q) circuit model, was normalized with respect to the baseline (R_Time_/R_Baseline_). The green line represents the baseline resistance, 10 min post‐pressure. Error bars represent the standard deviation of three technical replicates, analysed using an Ordinary one‐way Analysis of Variance: *p* ≤ 0.01 (**).

The fitted resistance of R_2_ from an R_1_(R_2_Q) circuit element is given as a fraction of the baseline resistance 10 min post‐pressure (Table S3, ESI provides the raw data). Representative data from three participants, one from each group (1–3) is shown (Figure 7) of the total thirteen (Figure S3, ESI).

**Group 1** R_2_ of Group 1 reduced over time, irrespective of the applied pressure. Furthermore, both skin sites displayed blanchable erythema. It is speculated that the frequent re‐application of electrodes disrupted the individuals' skin barrier, potentially due to the associated frictional and shear forces (*n* = 2).

**Group 2** showed a significant decrease in R_2_ at the pressure‐loaded site after 240 min (*p* ≤ 0.01) whilst the control region remained intact. Furthermore, only the pressure site had evidence of erythema. The Group 2 control arm (electrodes applied, no static pressure) showed no significant change in skin resistance or evidence of erythema (*n* = 3).

**Group 3** showed no change in R_2_ due to pressure; the average skin resistance over time does not drop below half of the baseline resistance and there was no erythema. These individuals were unaffected by static pressure (*n* = 8).

Overall, the impedance data shows that certain individuals are more sensitive to pressure than others. Where the skin resistance decreased, this was accompanied by blanchable erythema and is therefore indicative of disruption to the stratum corneum. The skin impedance protocol, using ECG electrodes, provides a useful tool to monitor an individual's sensitivity to mechanical loading.

The human volunteer study was not statistically powered, so absolute conclusions cannot be drawn. However, the apparent correlation between applied pressure and skin erythema suggests that using impedance measurements in this way – to monitor potential early‐stage pressure injury, may have utility in the long‐term monitoring of patients who are at risk of pressure injury. Examples include health conditions associated with immobility for example, a paralysed patient with a spinal injury, or which effect blood supply for example, a diabetic patient with neuropathy of the feet.^32^ A larger, properly powered clinical study would be needed to give confidence in these results. The information could lead to improvement on current pressure ulcer intervention strategies to better meet the needs of the individual, including when to offload the pressure site and selection of suitable pressure‐relieving devices.

## 5 CONCLUSIONS

In conclusion, this work demonstrates the potential utility of ECG electrodes combined with impedance spectroscopy as a method to measure changes in skin barrier function caused by pressure. Electrocardiogram electrodes are generally very cost‐effective and have good, conformable skin – electrode contact.^3^ Results suggest that skin impedance is much more sensitive than stratum corneum moisture content measurement at showing the effect of mechanical pressure loading on porcine skin (Figure 4 vs. Figure 6). Systematic thinning of the stratum corneum by tape stripping provided evidence that the method was predominately sensitive to the stratum corneum barrier function rather than deeper epidermal/dermal layers. This is an important distinction when considering the future development of a protocol which can monitor early‐stage pressure injury; early‐stage pressure ulceration visually presents as superficial skin damage. The measurement technique was also successfully applied in an ex vivo porcine static pressure model, showing clear reductions in fitted skin resistance when static pressure was applied owing to cellular deformation. Finally, a small study on how healthy human skin recovers from applied static pressure displayed inter‐individual variability; those who appeared more susceptible to pressure experienced blanchable erythema which was accompanied with reduced skin resistance. These preliminary results may provide some evidence that certain individuals are more prone to pressure injury than others. Furthermore, it may be possible to detect early‐stage pressure injuries in cases where there may be no visible damage, such as darker skin tones. Further clinical trials should be initiated on patients at risk of pressure injury to improve current intervention strategies and tailor them to the needs of the individual.

## CONFLICT OF INTEREST STATEMENT

None to declare.

## AUTHOR CONTRIBUTIONS

**Emily J. Owen**: Conceptualisation (Lead); Data curation (Lead); Formal analysis (Lead); Investigation (Lead); Methodology (Lead); Software (Lead); Writing – original draft (Lead); Writing – review & editing (Lead). **Hollie Hathaway**: Writing – review & editing (Supporting). **Bronwen Lafferty**: Writing – review & editing (Supporting). **A. Toby A. Jenkins**: Conceptualisation (Lead); Funding acquisition (Lead); Project administration (Lead); Supervision (Lead); Writing – original draft (Supporting); Writing – review & editing (Supporting).

## ETHICS STATEMENT

The University of Bath Research Ethics Committee have approved this study (EP 22 001).

## Supporting information

Supplementary Material

## Data Availability

The data that supports the findings of this study are available in the supplementary material of this article.
