# Supplementary material for: Using electrocardiogram electrodes to monitor skin impedance spectroscopic response when skin is subjected to sustained static pressure
Source: Skin Health Dis. 2023 Mar 6;3(4):e225. doi: 10.1002/ski2.225 (PMC10395637; doi:10.1002/ski2.225)
Supplement: Supplementary file 1 — Supplementary Material [file SKI2-3-e225-s001.docx]

**Electronic Supporting Information**

| **Table S1.** Changes in circuit elements, based on the R_1_(R_2_Q) equivalent circuit model, when *ex vivo* porcine skin is tape stripped up to 110 times. | | | | |
| --- | --- | --- | --- | --- |
| Number of Tape Strips | R_1_ / Ω | R_2_ / Ω | Q / µT | n / ɸ |
| 0 | 4.8E+01  9.9E+01  5.1E+01 | 2.1E+05  1.4E+05  5.9E+05 | 0.10  0.07  0.06 | 0.73  0.74  0.74 |
| 10 | 1.7E+02  2.0E+02  2.1E+02 | 9.5E+04  2.1E+05  2.8E+05 | 0.12  0.08  0.09 | 0.73  0.75  0.74 |
| 30 | 3.4E+02  3.6E+02  4.4E+02 | 1.6E+03  4.6E+03  7.8E+03 | 0.25  0.10  0.11 | 0.77  0.80  0.77 |
| 50 | 3.4E+02  3.6E+02  4.5E+02 | 5.1E+02  7.9E+02  1.5E+03 | 0.31  0.10  0.18 | 0.79  0.83  0.77 |
| 70 | 3.5E+02  3.5E+02  4.5E+02 | 3.2E+02  4.1E+02  8.5E+02 | 0.57  0.27  0.20 | 0.73  0.77  0.77 |
| 90 | 2.7E+02  3.6E+02  4.3E+02 | 3.0E+02  3.9E+02  8.7E+02 | 1.37  0.19  0.19 | 0.62  0.79  0.79 |
| 110 | 2.7E+02  3.5E+02  4.2E+02 | 2.8E+02  3.9E+02  6.2E+02 | 1.82  0.25  0.23 | 0.60  0.77  0.78 |
|  | | | | |

| **Table S2.** Changes in circuit elements, based on the R_1_(R_2_Q) equivalent circuit model, when *ex vivo* porcine skin is subjected to static pressure of up to 22.5 mmHg for 24 hours. | | | | | |
| --- | --- | --- | --- | --- | --- |
| Time / h | Pressure / mmHg | R_1_ / Ω | R_2_ / Ω | Q / µT | n / ɸ |
| 0 | 0.0 | 1.77E+02  1.09E+02  5.57E+01  1.20E+02 | 2.40E+05  1.41E+05  5.80E+05  8.21E+05 | 0.08  0.09  0.06  0.05 | 0.76  0.74  0.76  0.77 |
|  | 7.5 | 1.47E+02  9.61E+00  2.00E+02  9.51E+01 | 4.65E+05  3.88E+05  2.37E+05  1.05E+06 | 0.08  0.07  0.04  0.04 | 0.75  0.73  0.79  0.78 |
|  | 15.0 | 2.05E+02  Out of Range  5.00E+01  Out of Range | 9.01E+04  5.81E+05  8.53E+05  1.31E+06 | 0.13  0.11  0.05  0.05 | 0.72  0.70  0.75  0.75 |
|  | 22.5 | 7.49E+00  Out of Range  1.14E+01  Out of Range | 1.72E+06  1.66E+06  3.37E+05  1.83E+06 | 0.05  0.05  0.04  0.04 | 0.75  0.75  0.77  0.76 |
| 2 | 0.0 | 2.58E+02  2.43E+02  3.25E+02  1.49E+02 | 2.66E+05  1.49E+05  8.81E+05  1.60E+06 | 0.07  0.09  0.06  0.05 | 0.76  0.75  0.76  0.78 |
|  | 7.5 | 1.51E+02  1.70E+02  Out of Range  1.13E+02 | 6.09E+05  2.69E+05  6.23E+05  2.11E+06 | 0.07  0.06  0.09  0.04 | 0.75  0.76  0.71  0.77 |
|  | 15.0 | 1.31E+02  Out of Range  8.11E+01  1.14E+02 | 1.59E+05  6.54E+05  1.11E+06  1.53E+06 | 0.10  0.11  0.06  0.06 | 0.73  0.69  0.75  0.75 |
|  | 22.5 | 1.58E+02  8.38E+01  1.00E-06  8.22E+01 | 1.53E+06  1.72E+06  1.64E+06  1.98E+06 | 0.06  0.07  0.06  0.05 | 0.75  0.75  0.74  0.76 |
| 4 | 0.0 | 1.34E+02  1.75E+02  5.75E+01  2.49E+02 | 3.47E+05  1.47E+05  1.40E+06  1.84E+06 | 0.07  0.09  0.05  0.04 | 0.77  0.75  0.76  0.79 |
|  | 7.5 | 1.32E+02  1.53E+02  1.02E+02  1.67E+02 | 8.50E+05  3.22E+05  3.53E+05  1.53E+06 | 0.06  0.06  0.06  0.05 | 0.75  0.76  0.75  0.77 |
|  | 15.0 | 2.26E+02  1.00E-06  1.38E+02  9.24E+01 | 3.86E+05  6.31E+05  6.45E+05  1.09E+06 | 0.05  0.12  0.06  0.06 | 0.77  0.69  0.76  0.75 |
|  | 22.5 | 1.75E+02  1.32E+02  1.25E+02  1.20E+02 | 1.42E+06  1.39E+06  1.31E+06  1.97E+06 | 0.06  0.07  0.06  0.05 | 0.76  0.75  0.76  0.77 |
| 6 | 0.0 | 8.84E+01  1.48E+02  1.19E+02  2.02E+02 | 4.05E+05  3.79E+05  1.26E+06  1.78E+06 | 0.07  0.07  0.05  0.05 | 0.76  0.77  0.77  0.78 |
|  | 7.5 | 1.20E+02  2.11E+02  1.00E-06  1.39E+02 | 9.34E+05  3.09E+05  8.76E+05  1.53E+06 | 0.05  0.05  0.09  0.05 | 0.76  0.77  0.71  0.77 |
|  | 15.0 | 2.06E+01  1.00E-06  1.00E-06  1.65E+02 | 2.51E+05  5.26E+05  1.11E+06  6.63E+05 | 0.10  0.09  0.07  0.04 | 0.72  0.72  0.73  0.78 |
|  | 22.5 | 1.52E+02  1.30E+02  6.63E+00  1.93E+02 | 1.31E+06  1.36E+06  9.48E+05  1.15E+06 | 0.06  0.07  0.07  0.04 | 0.77  0.76  0.74  0.79 |
| 12 | 0.0 | 1.39E+02  1.56E+02  1.29E+02  2.10E+02 | 2.29E+05  1.67E+05  6.12E+05  1.73E+06 | 0.08  0.11  0.06  0.05 | 0.76  0.74  0.77  0.78 |
|  | 7.5 | 1.46E+02  1.66E+02  Out of Range  2.06E+02 | 9.38E+05  4.11E+05  9.39E+05  9.78E+05 | 0.05  0.06  0.09  0.05 | 0.77  0.76  0.71  0.77 |
|  | 15.0 | 1.93E+01  1.07E+01  9.16E+02  4.29E+02 | 3.60E+05  4.87E+05  1.29E+06  5.96E+05 | 0.09  0.09  0.06  0.04 | 0.72  0.72  0.74  0.77 |
|  | 22.5 | 1.90E+02  2.11E+03  6.85E+01  Out of Range | 1.12E+06  9.36E+05  6.20E+05  5.54E+05 | 0.06  0.06  0.07  0.10 | 0.77  0.77  0.74  0.71 |
| 24 | 0.0 | 1.23E+02  1.43E+02  3.64E+01  2.10E+02 | 4.14E+05  2.78E+05  4.82E+05  1.46E+06 | 0.07  0.09  0.08  0.05 | 0.76  0.75  0.73  0.78 |
|  | 7.5 | 1.90E+02  2.01E+02  3.40E+02  1.87E+02 | 2.26E+05  1.50E+05  2.06E+05  2.96E+05 | 0.08  0.09  0.07  0.07 | 0.77  0.76  0.76  0.75 |
|  | 15.0 | 1.58E+02  1.72E+02  6.52E+02  4.46E+02 | 1.26E+05  1.23E+05  1.53E+05  2.56E+05 | 0.10  0.10  0.08  0.06 | 0.74  0.74  0.77  0.77 |
|  | 22.5 | 3.16E+02  3.05E+02  2.90E+02  1.72E+02 | 1.84E+05  1.59E+05  1.25E+05  2.06E+05 | 0.07  0.09  0.08  0.09 | 0.77  0.75  0.77  0.73 |
|  | | | | | |

| **Table S3.** Changes in circuit elements, based on the R_1_(R_2_Q) equivalent circuit model, up to 240 mins after *in vivo* human skin is subjected to 88 mmHg of static pressure. | | | | | |
| --- | --- | --- | --- | --- | --- |
| Group | Participant | R_1_ / Ω | R_2_ / Ω | Q / µT | n / ɸ |
| Pressure Site - 10 mins After Mechanical Loading (Baseline) | | | | | |
| 1 | 1 | 2.34E+02  3.04E+02  3.05E+02 | 1.60E+06  2.51E+06  5.77E+05 | 0.06  0.06  0.08 | 0.86  0.87  0.86 |
|  | 2 | 2.37E+02 | 7.06E+05 | 0.08 | 0.85 |
|  | 3 | 2.88E+02 | 2.47E+06 | 0.05 | 0.88 |
| 2 | 4 | 3.11E+02  3.28E+02  2.88E+02 | 7.63E+05  1.08E+06  1.16E+06 | 0.05  0.06  0.07 | 0.86  0.85  0.84 |
|  | 5 | 2.38E+02 | 9.05E+05 | 0.09 | 0.84 |
| 3 | 6 | 3.55E+02 | 3.66E+06 | 0.05 | 0.87 |
|  | 7 | 3.49E+02  3.71E+02  3.16E+02 | 1.03E+07  9.17E+06  5.43E+06 | 0.03  0.02  0.03 | 0.88  0.90  0.88 |
|  | 8 | 3.36E+02 | 4.47E+06 | 0.05 | 0.89 |
|  | 9 | 3.19E+02 | 2.09E+06 | 0.06 | 0.88 |
|  | 10 | 2.23E+02 | 2.89E+06 | 0.07 | 0.86 |
|  | 11 | 3.30E+02 | 1.94E+06 | 0.03 | 0.88 |
|  | 12 | 3.32E+02 | 4.35E+06 | 0.06 | 0.87 |
|  | 13 | 3.45E+02 | 2.22E+06 | 0.07 | 0.87 |
| Control Site - 10 mins After Mechanical Loading (Baseline) | | | | | |
| 1 | 1 | 2.15E+02  2.79E+02  2.66E+02 | 1.79E+06  2.20E+06  1.15E+06 | 0.07  0.07  0.07 | 0.85  0.86  0.86 |
|  | 2 | 2.56E+02 | 1.04E+06 | 0.09 | 0.85 |
|  | 3 | 2.75E+02 | 2.26E+06 | 0.06 | 0.88 |
| 2 | 4 | 3.33E+02  3.12E+02  2.82E+02 | 2.43E+06  2.89E+06  2.78E+06 | 0.05  0.05  0.05 | 0.87  0.85  0.85 |
|  | 5 | 2.35E+02 | 1.53E+06 | 0.08 | 0.84 |
| 3 | 6 | 3.45E+02 | 4.13E+06 | 0.05 | 0.86 |
|  | 7 | 3.13E+02  3.62E+02  3.57E+02 | 1.25E+07  1.25E+07  6.26E+06 | 0.03  0.02  0.03 | 0.89  0.89  0.89 |
|  | 8 | 3.25E+02 | 5.76E+06 | 0.05 | 0.88 |
|  | 9 | 3.34E+02 | 5.12E+06 | 0.06 | 0.88 |
|  | 10 | 2.22E+02 | 2.17E+06 | 0.06 | 0.87 |
|  | 11 | 3.19E+02 | 4.52E+06 | 0.04 | 0.88 |
|  | 12 | 3.07E+02 | 2.76E+06 | 0.06 | 0.88 |
|  | 13 | 3.73E+02 | 4.61E+06 | 0.06 | 0.86 |
| Pressure Site - 240 mins After Mechanical Loading | | | | | |
| 1 | 1 | 2.49E+02  2.81E+02  2.77E+02 | 3.31E+05  7.89E+05  2.67E+05 | 0.06  0.06  0.10 | 0.87  0.87  0.84 |
|  | 2 | 2.59E+02 | 2.46E+05 | 0.08 | 0.85 |
|  | 3 | 2.67E+02 | 2.00E+05 | 0.07 | 0.86 |
| 2 | 4 | 3.10E+02  3.47E+02  3.38E+02 | 3.29E+04  1.10E+05  1.10E+05 | 0.07  0.05  0.07 | 0.84  0.89  0.86 |
|  | 5 | 2.33E+02 | 2.608 | 0.10 | 0.84 |
| 3 | 6 | 3.71E+02 | 4.72E+06 | 0.05 | 0.86 |
|  | 7 | 3.71E+02  4.13E+02  3.28E+02 | 1.44E+07  1.54E+07  5.25E+06 | 0.03  0.02  0.03 | 0.88  0.89  0.86 |
|  | 8 | 2.77E+02 | 5.38E+06 | 0.05 | 0.88 |
|  | 9 | 2.93E+02 | 2.34E+06 | 0.07 | 0.86 |
|  | 10 | 2.47E+02 | 1.89E+06 | 0.07 | 0.86 |
|  | 11 | 2.97E+02 | 8.31E+05 | 0.04 | 0.88 |
|  | 12 | 2.74E+02 | 3.66E+06 | 0.07 | 0.87 |
|  | 13 | 3.22E+02 | 2.68E+06 | 0.07 | 0.86 |
| Control Site - 240 mins After Mechanical Loading | | | | | |
| 1 | 1 | 2.42E+02  2.98E+02  2.75E+02 | 6.37E+05  3.90E+05  6.95E+04 | 0.07  0.07  0.14 | 0.85  0.87  0.82 |
|  | 2 | 2.83E+02 | 3.77E+05 | 0.09 | 0.86 |
|  | 3 | 3.13E+02 | 1.69E+05 | 0.06 | 0.87 |
| 2 | 4 | 3.35E+02  2.99E+02  2.68E+02 | 2.46E+06  1.91E+06  1.49E+06 | 0.05  0.06  0.07 | 0.86  0.85  0.84 |
|  | 5 | 2.46E+02 | 1.52E+06 | 0.08 | 0.85 |
| 3 | 6 | 3.42E+02 | 5.11E+06 | 0.05 | 0.86 |
|  | 7 | 3.23E+02  3.69E+02  2.57E+02 | 1.23E+07  1.30E+07  1.71E+06 | 0.03  0.02  0.04 | 0.88  0.88  0.85 |
|  | 8 | 2.62E+02 | 2.75E+06 | 0.07 | 0.85 |
|  | 9 | 3.00E+02 | 3.17E+06 | 0.07 | 0.86 |
|  | 10 | 2.25E+02 | 2.70E+06 | 0.06 | 0.87 |
|  | 11 | 2.82E+02 | 3.75E+06 | 0.05 | 0.88 |
|  | 12 | 2.99E+02 | 3.09E+06 | 0.07 | 0.87 |
|  | 13 | 3.75E+02 | 3.14E+06 | 0.07 | 0.85 |
|  | | | | | |

| **A** | **B** | **C** |
| --- | --- | --- |
| **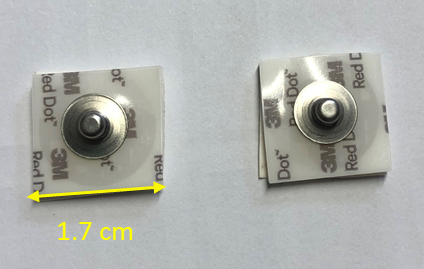** | **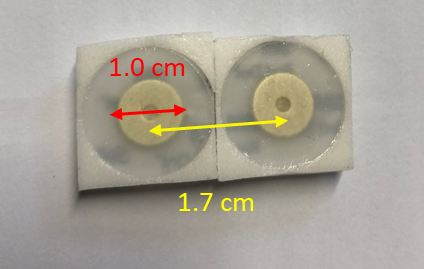** | **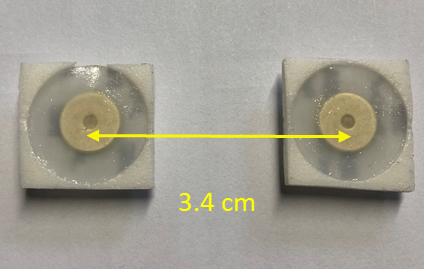** |
| **Figure S1.** Red Dot 3M electrodes: pre-gelled disposable Ag / AgCl electrodes surrounded by an adhesive border, used for skin impedance measurements. The electrode devices are trimmed to 1.7 cm by 1.7 cm, including the adhesive border (A). The electrodes within the devices have a diameter of 1 cm and the distance between the centre of each electrode for *ex vivo* porcine skin measurements is 1.7 cm (B) and the distance between them is 3.4 cm for *in vivo* human skin (C). | | |

| **A** | **B** |
| --- | --- |
| **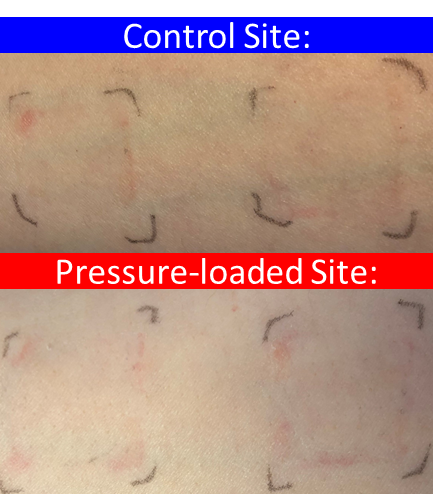** | **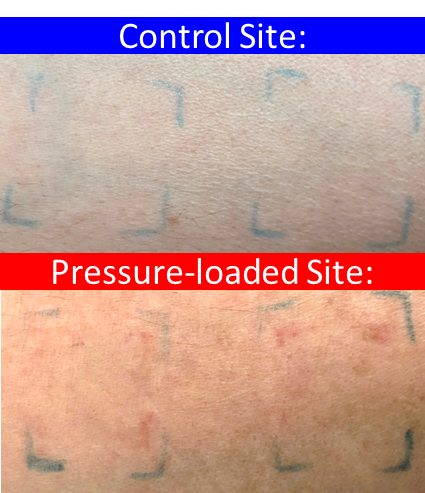** |
| **C** | **D** |
| 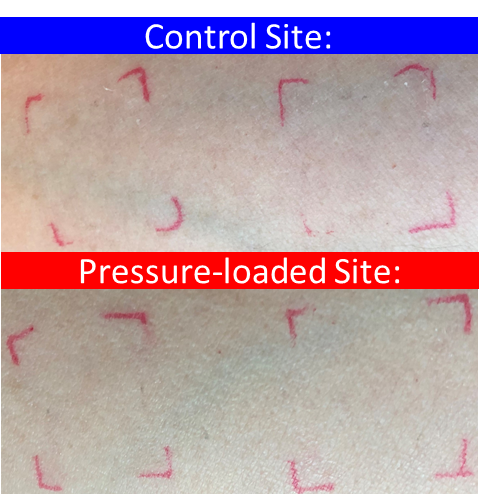 | 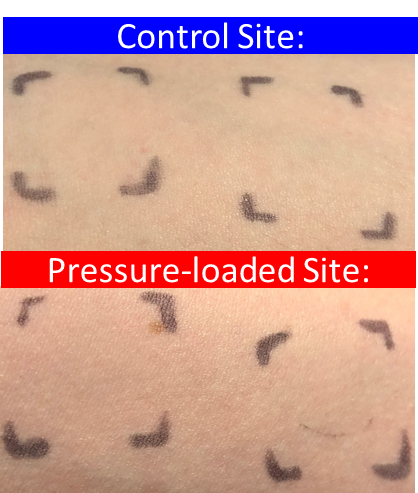 |
| **Figure S2.** Macroscopic images of for *in vivo* human ventral forearm skin of four participants (A-D), up to 240 mins after applying 88 mmHg of static pressure. | |

| **A (Group 1)** |
| --- |
| **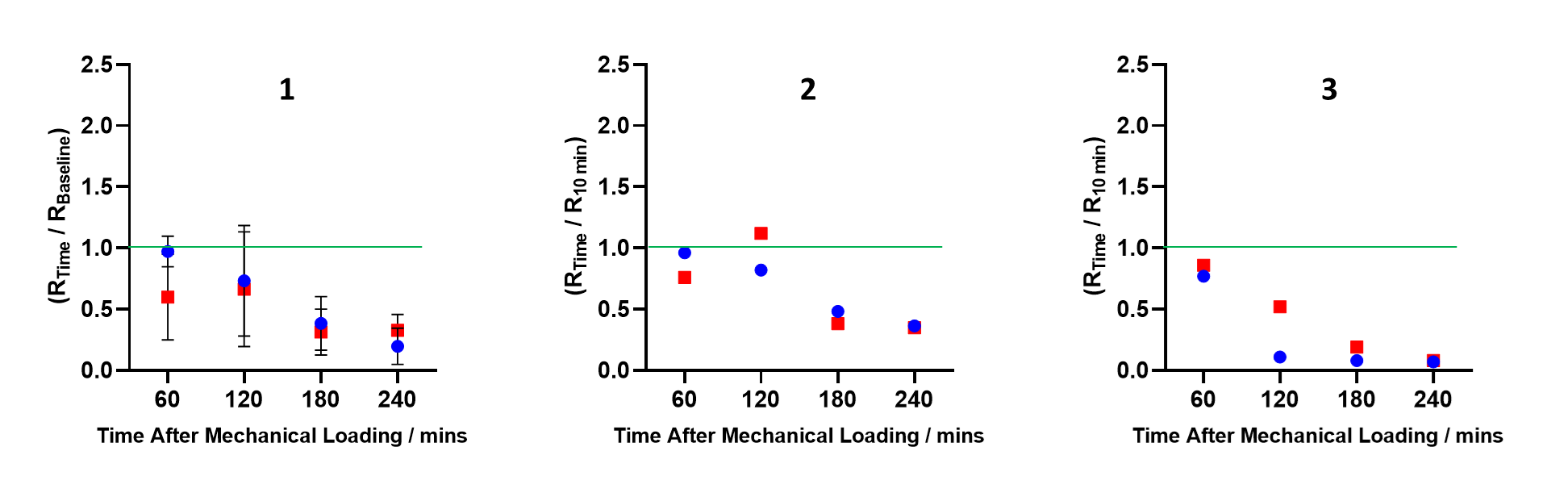** |
| **B (Group 2)** |
| **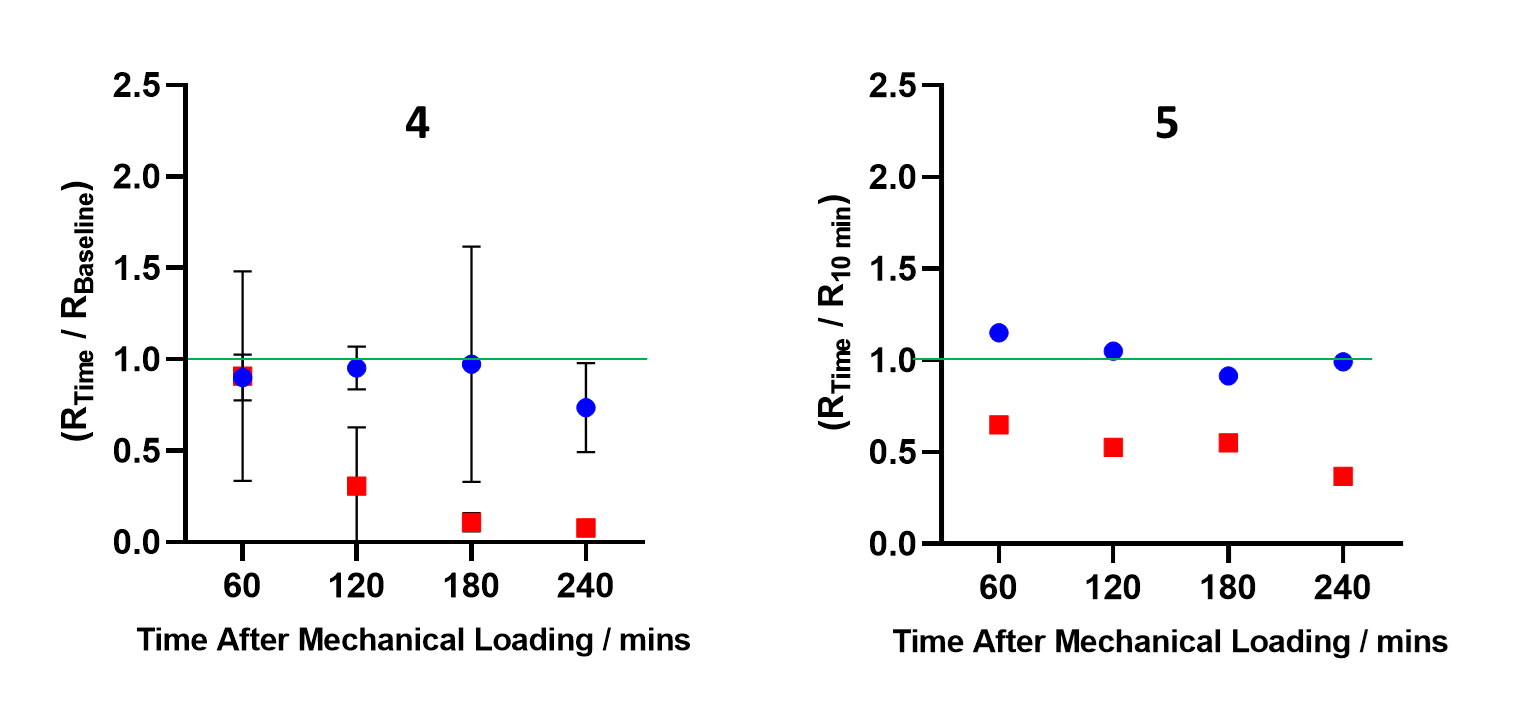** |
| **C (Group 3)** |
| **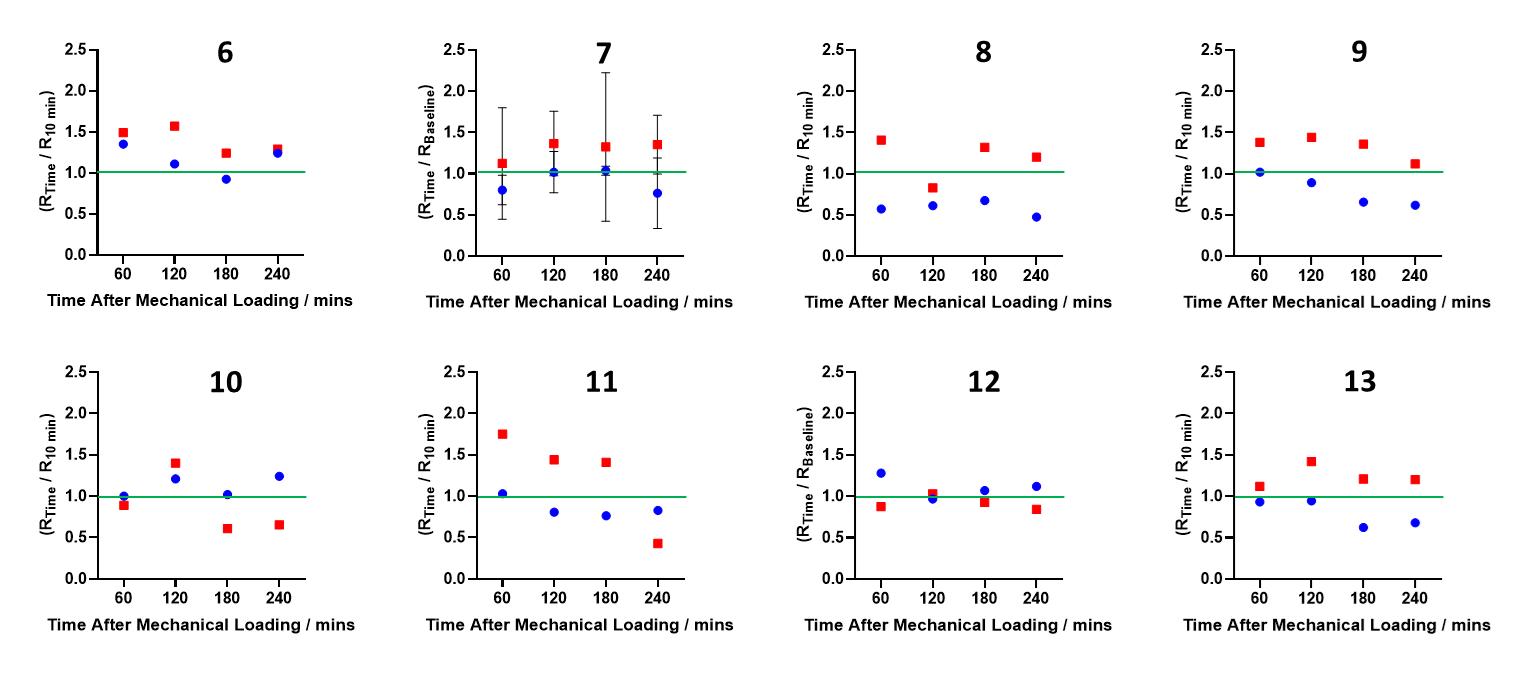** |
|  |
| **Figure S3.** Impedance data for *in vivo* human ventral forearm skin, up to 240 mins after applying 88 mmHg of static pressure. Fitted resistance ‘R_2_’, from an R_1_(R_2_Q) circuit model, was normalised with respect to the baseline (R_Time_ / R_Baseline_). Data is divided into three groups: decrease in R_2_ of control and pressure site (A – Group 1), decrease in R2 of pressure site (B – Group 2) and no decrease in R_2_ (C – Group 3). Error bars, where given, represent the standard deviation of three technical replicates measured on the same site but at a different date. |
